# Quality of Life and Body Dissatisfaction in Cisgender Men Reporting Sexual Attraction Toward Men or Women

**DOI:** 10.5964/ejop.11423

**Published:** 2024-08-30

**Authors:** Liam Cahill, Joost M. Leunissen, Mike Marriott, Andrew K. Dunn

**Affiliations:** 1 Department of Psychology, School of Social Sciences, Nottingham Trent University, Nottingham, United Kingdom; 2 Department of Psychology, University of Winchester, Winchester, United Kingdom; Victoria University of Wellington, Wellington, New Zealand

**Keywords:** body dissatisfaction, quality of life, sexual attraction, sexual identity

## Abstract

Gay men report poorer body image than straight men, but no research has examined other dimensions of sexual identity (e.g., sexual attraction). Most research also focused on clinical outcomes of body dissatisfaction rather than subclinical influences on quality of life. We examined the association between sexual attraction (i.e., attraction to men or women), body dissatisfaction and quality of life in cisgender men. We hypothesised that: 1) men attracted to men would report higher body dissatisfaction, 2) men attracted to men would report lower quality of life (i.e., psychological, physiological, social, and environmental), 3) body dissatisfaction would be negatively associated with quality of life, and finally 4) body dissatisfaction mediates the association between sexual attraction and quality of life. A cross-sectional study (86 men attracted to men and 87 men attracted to women) supported these hypotheses but showed that sexual attraction was only associated with psychological quality of life. While sexual attraction was only associated with psychological quality of life, there were indirect associations with all quality of life domains acting through body dissatisfaction. Our findings emphasise that men attracted to men are at greater risk of poor body image and that body dissatisfaction is a pervasive health problem, negatively influencing subclinical health and well-being in cisgender men. We also highlight how body dissatisfaction may be one psychological process negatively influencing the psychological quality of life in men attracted to men. More resources should be directed toward preventing body dissatisfaction, particularly in sexual minoritised men.

Body dissatisfaction (a dislike for one’s bodily appearance; Heider et al., 2018) is an important research area because increased body dissatisfaction is associated with detrimental physical (e.g., restrictive eating) and psychological (e.g., depression) outcomes (Barnes et al., 2020; Blashill, 2011). Research indicates that body dissatisfaction has subclinical adverse effects by impairing an individual’s quality of life (Duarte et al., 2015; Santhira Shagar et al., 2021). In Western societies, body dissatisfaction is normative and widespread (Tantleff-Dunn et al., 2011) and is argued to be a pervasive public health problem (Griffiths et al., 2017; Rodgers et al., 2023). Research addressing the influences of body dissatisfaction on quality of life has mainly focused on women or on examining a narrow definition of quality of life in men (e.g., psychological quality of life only; Austen et al., 2020; Griffiths et al., 2016; Santhira Shagar et al., 2021). Limited research has explored a broad conceptualisation of quality of life in cisgender men^1^1We define cisgender as “*people who do not identify as trans or who identify with the sex they were assigned at birth*” (McDermott et al., 2018, p. 69)..

Additionally, while research has illustrated that gay men systematically experience greater body dissatisfaction relative to straight (i.e., heterosexual) men (Dahlenburg et al., 2020), no research to date has explored other aspects of sexual identity. Sexual identity is multidimensional (Kaestle, 2019; Savin-Williams, 2016), comprising a self-defined identity (i.e., gay or straight), sexual attraction (i.e., attraction to the same or different gender^2^2We deemed it appropriate to define cisgender men who self-reported sexual attraction to other men as expressing *same-gender attraction* and men who reported attraction to women as expressing *different-gender attraction*. We used *different gender attraction* to be inclusive and not to endorse a binary view of gender (i.e., same vs opposite).) and sexual behaviour (i.e., with whom they have has sexual contact). People vary along each of these dimensions. For instance, a man may identify as straight but report attraction towards and engage in sexual behaviours with men. Despite this, no research has examined whether dimensions of sexual identity other than self-reported identity, such as self-reported sexual attraction, are associated with men’s body dissatisfaction or quality of life. Nor has anyone examined whether body dissatisfaction might mediate any association between sexual attraction and quality of life in a sample of cisgender men (henceforth referred to as men for simplicity).

## Sexual Identity and Body Dissatisfaction

Research indicates that self-identifying gay men generally have greater body dissatisfaction than straight men (Dahlenburg et al., 2020). Gay men are more likely to endorse societal body ideals and emphasise having an ideal body type, exacerbating body dissatisfaction (Brewster et al., 2017). Moreover, gay men experience increased pressures from within gay communities to have an ideal body in which a lean and muscular body type is endorsed and idealised (Tylka & Andorka, 2012). While previous research has established that gay men experience greater body dissatisfaction, no previous research has explored the associations between other elements of sexual identity and body dissatisfaction, such as self-reported sexual attraction (He et al., 2020). To fully understand the unique experiences that sexual minoritised populations may experience concerning their body image and how they navigate this, it is important to examine other aspects of sexual identity (e.g., sexual attraction).

Men attracted to men likely experience poorer body image than men attracted to women for several reasons. Men who report non-heterosexual (i.e., straight) attraction are a minoritised group, according to Minority Stress Theory (Meyer, 2003). People in a minoritised population experience a range of adverse psychological outcomes, including greater body dissatisfaction (Rodgers et al., 2023). Men deviating from heteronormative ideals may fear appearance-based discrimination. They often attempt to conceal their minoritised status by adhering to traditional masculine ideals by changing their bodies (Brewster et al., 2017). This may cause greater body dissatisfaction, mainly as those masculine ideals are challenging to attain. For example, developing muscles is incompatible with pursuing a lean physique, making the community’s ideal body type challenging to attain (Tiggemann et al., 2007).

Further, men attracted to men are also likely to experience greater objectification. Objectification Theory (Fredrickson & Roberts, 1997) posits that societal members who are frequently sexualised and evaluated based on their physical appearance may internalise those evaluations and objectify themselves (i.e., self-objectification), increasing body dissatisfaction (Brewster et al., 2017). For example, within gay communities, physical attractiveness is crucial in consolidating romantic and sexual interest (Frederick & Essayli, 2016; Tylka & Andorka, 2012). Gay men are subjected to evaluations from other men, termed the *male gaze* (Wood, 2004). As men who report attraction to other men experience similar pressures, we expect a similar increase in self-objectification and body dissatisfaction.

## Body Dissatisfaction and Quality of Life

Previous investigations assessing the consequences of body dissatisfaction have primarily focused on mood disorders (Paxton et al., 2006) or eating and exercise psychopathologies, such as disordered eating (Kaminski et al., 2005). Although it is crucial to establish associations between body dissatisfaction and psychopathology, this focus is too narrow. Body dissatisfaction may not always result in clinically diagnosable symptoms, and the absence of a clinical diagnosis does not negate the negative influence of body dissatisfaction on well-being and psychological functioning. To get a comprehensive understanding of the association between body dissatisfaction and well-being, we focused on quality of life, which is a subclinical indicator of well-being.

Quality of life encompasses a person’s psychological (e.g., their mental health), physiological (e.g., health complaints), social (e.g., the quality of their social relationships), and environmental (e.g., the general safety and volatility of a person’s immediate environment) well-being (Skevington et al., 2004). Increased body dissatisfaction is associated with decreased psychological, social, and physiological quality of life in women (Mond et al., 2013) and adolescent boys and girls (Griffiths et al., 2017). Research has also demonstrated that increased body dissatisfaction in women is associated with each domain of quality of life (Santhira Shagar et al., 2021). While a similar pattern has emerged in men in some specific domains, such as psychological and physiological quality of life (Griffiths et al., 2016, 2018, 2019), no research has comprehensively examined how body dissatisfaction is associated with quality of life across each domain in men. This is important as incidence rates of body dissatisfaction in men remain high, and elucidating potential subclinical consequences is paramount.

Body dissatisfaction is a global public health concern due to poor body image’s wide-reaching individual and societal impacts (Rodgers et al., 2023). It is likely that body dissatisfaction negatively influences a person’s quality of life in several ways. Greater dissatisfaction with one’s body causes psychological distress, which may reduce psychological quality of life by lowering self-esteem and promoting negative affect (Barnes et al., 2020; Blashill, 2011). Body dissatisfaction also stimulates behaviour that changes one’s appearance, such as unhealthy dieting and excessive exercise, impairing physical health and lowering a person’s physiological quality of life (Kaminski et al., 2005). Equally, these behaviours may also influence psychological quality of life.

Heightened body dissatisfaction can negatively impact relationships. Under the Tripartite Influence Model (or Quadripartite Influence Model for men), the media, friends, family, and partners can be substantial sources of pressure to be attractive and to adopt an ideal body type (Tylka, 2011). These pressures may increase body dissatisfaction and cause men to view their close relationships (i.e., family, friends, and partners) as sources of pressure and judgment. Further, poor body image may lead to heightened competition and appearance-related comparisons among peers and friendship groups (Lawler & Nixon, 2011).

Environmental quality of life may equally be impaired, as body dissatisfaction may create a hostile and confrontational environment, marked by the belief that one’s body is being observed, objectified, and judged (Brewster et al., 2017; Santhira Shagar et al., 2021). Further, according to sociocultural theories of body dissatisfaction, appearance-related messages (e.g., unrealistically muscular ideals) from society contribute to poor body image (Tylka, 2011). Navigating the messages around body image in media (e.g., social media) and the discourse surrounding appearance-related messages may cause people to view their broader environmental quality of life poorly. These findings emphasise how body dissatisfaction may reduce a person’s quality of life across each domain.

## Sexual Identity and Quality of Life

To summarise, there are several reasons why body dissatisfaction would reduce quality of life across each domain in men. Similarly, men attracted to men likely experience a range of unique experiences and stressors that directly negatively influence each quality of life domain. For example, previous research demonstrates that sexual minoritised men experience a lower quality of life in specific domains, such as health and sexual quality of life (Grabski et al., 2019; Wen & Zheng, 2019). However, limited research explores the relationship between self-reported sexual attraction and quality of life across psychological, physiological, social, and environmental domains.

Nevertheless, there is reason to posit that members of society occupying a minoritised status would generally experience lower quality of life. Minority Stress Theory (Meyer, 2003) suggests that those who occupy a minoritised status (e.g., sexual minoritised men) are at risk of poorer mental health, including depression and suicidality (e.g., Marshal et al., 2011). Similarly, sexual minoritised men experience increased stigmatisation and marginalisation within society (Grabski et al., 2019), experience greater physiological complications such as HIV and sexually transmitted infections (Cochran & Mays, 2007; Remor, 2002), and are at higher risk of violent assault, victimisation, and even death (Chen et al., 2020; ILGA, 2020). These factors illustrate potential avenues for reduced quality of life across each domain in minoritised men, including men who report attraction to men.

## Body Dissatisfaction as a Mediator

Men attracted to men may experience reduced quality of life for several reasons. However, to our knowledge, no previous research has addressed whether body dissatisfaction may be a psychological process that mediates the association between self-reported attraction and quality of life. Given that men attracted to men may experience uniquely heightened body dissatisfaction and the potentially detrimental effects of body dissatisfaction on quality of life across each domain, it is prudent to address whether body dissatisfaction mediates the association between sexual identity (specifically sexual attraction) and quality of life.

## The Current Research

Previous research has shown that gay, relative to straight men, experience greater body dissatisfaction (Dahlenburg et al., 2020). Whilst this research is valuable, it is necessary to explore other aspects of sexual identity. Most research has also focused on clinical outcomes of body dissatisfaction, but body dissatisfaction also decreases subclinical health and well-being (Griffiths et al., 2016). Research across each domain of quality of life (i.e., psychological, physiological, social and environmental) has focused on women and only a restricted number of domains have been explored in men (Griffiths et al., 2016; Santhira Shagar et al., 2021). Moreover, while research has illustrated that sexual minoritised men are at greater risk of poorer quality of life, no research has explored the association between the sexual attraction component of sexual identity and quality of life across each domain. Finally, while previous research has established a range of potential reasons why men attracted to men may experience poorer quality of life, no research to date has explored whether body dissatisfaction is one such psychological process.

Given the health concerns surrounding body dissatisfaction, understanding what predicts poorer body image is important (Rodgers et al., 2023). More targeted interventions can be developed by identifying groups most at risk of body dissatisfaction. Identifying the potential quality of life repercussions linked to body dissatisfaction in men is also crucial. These insights will contribute to the ongoing discourse concerning the potential health concerns associated with greater body dissatisfaction. Our findings will also have theoretical implications, furthering our understanding of what aspects of sexual identity are associated with body dissatisfaction and quality of life and whether body dissatisfaction is one psychological process that might explain the potentially lower quality of life in men attracted to men.

**Figure 1:**
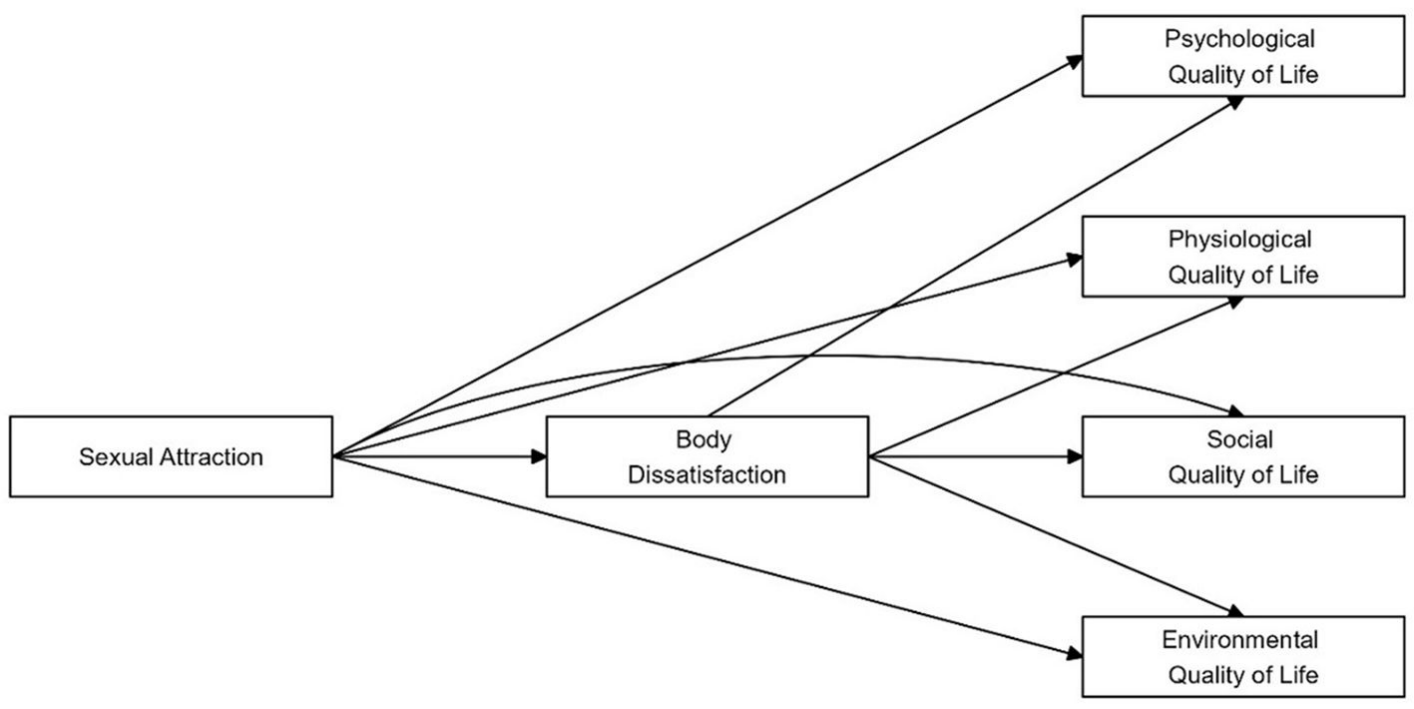
The Mediation Model

In summary, this research focuses on men’s self-reported sexual attraction toward men and women. We also explore whether sexual attraction and body dissatisfaction are associated with quality of life across each domain (i.e., psychological, physiological, social and environmental) and whether body dissatisfaction is one process that might mediate the association between sexual attraction and quality of life. Figure 1 illustrates our model. We hypothesised that:

**Hypothesis 1**: Men attracted to men report greater body dissatisfaction than men attracted to women.

**Hypothesis 2**: Men attracted to men report lower quality of life across each domain (i.e., psychological, physiological, social, and environmental) than men attracted to women.

**Hypothesis 3**: Higher body dissatisfaction is negatively associated with quality of life (i.e., psychological, physiological, social, and environmental).

**Hypothesis 4**: Body dissatisfaction mediates the association between sexual attraction and quality of life.

## Method

### Participants and Design

This study adopted an online correlational design. We recruited 173 participants who identified as cisgender men through the crowdsourcing platform Prolific (*n* = 154) and our psychology department’s participant recruitment scheme, which rewards students for participating in departmental research by allowing them to use the scheme to recruit participants for their projects (*n* = 19). As culture influences body ideals (Paxton et al., 2006), we restricted participation to UK and Ireland residents. We removed nine participants who indicated they did not reside in the UK or Ireland. The sample was majority White (*n* = 168; 97.1%) and aged between 18 and 60 (*M* = 33.6, *SD* = 12.0). We categorised our sample (see below) into 86 men who reported attraction predominantly to men (*M_age_* = 31.2, *SD_age_* = 11.3) and 87 men who reported attraction predominantly to women (*M*_age_ = 36.0, *SD_age_* = 12.2). Ethical approval for this study was provided by the ethics board at Nottingham Trent University. A sensitivity power analysis indicated that our study was powered to detect associations of *r* = .21 (or Cohen’s *d* = 0.43) and higher (power = .80, alpha = .05).

### Materials and Procedure

Our participants read an information sheet and provided informed consent. Next, the participants completed the following measures in this order (see Table 1 for descriptive statistics).

**Table 1:** Descriptive Statistics and Correlations

Measure	*M*	*SD*	Range	α	1	2	3	4	5
1. MBAS	101.65	28.63	24–161	.93	—	-.41	-.32	-.32	-.32
2. Psychological QoL	26.87	7.30	7–42	.85	-.53, -.28	—	.61	.72	.75
3. Physiological QoL	38.46	7.77	14–49	.83	-.44, -.17	.51, .70	—	.45	.58
4. Social QoL	13.21	4.55	3–21	.78	-.45, -.18	.64, .78	.32, .56	—	.62
5. Environmental QoL	40.60	8.83	10–56	.87	-.45, -.18	.67, .81	.47, .58	.52, .70	—

*Note.* QoL: quality of life. Range is the observed range in the data. Pearson’s *r* above the diagonal, 95% confidence intervals below the diagonal.

All correlations were significant at *p* < .05.

#### Sexual Attraction

**Figure 2:**
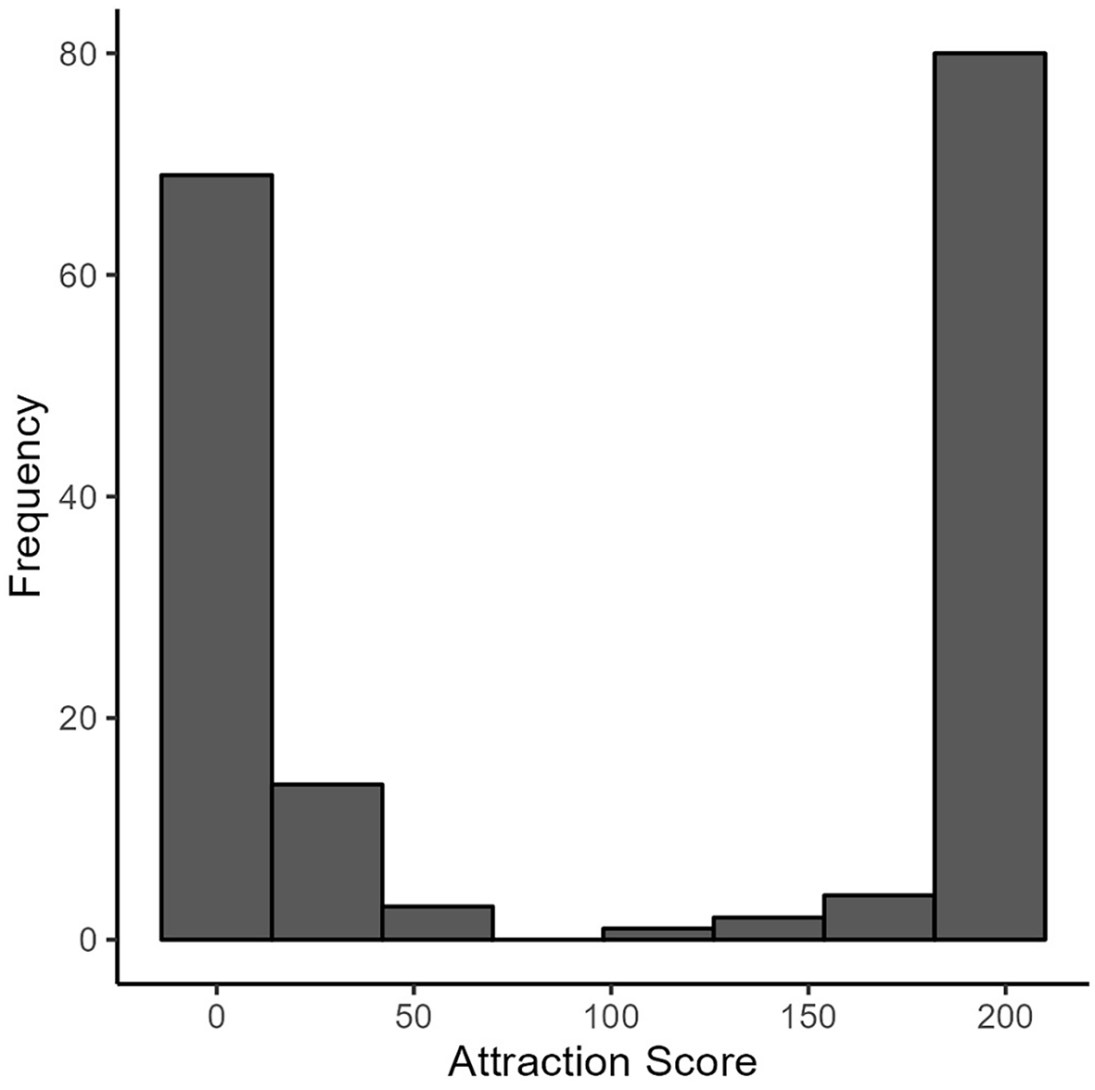
Histogram of Attraction Measure Scores

We captured sexual attraction as an indicator of sexual identity (Kaestle, 2019). Consistent with previous approaches (e.g., Morgenroth et al., 2022), our participants reported their sexual attraction toward (1) women and (2) men on two continuous scales: 1–*not at all attracted to*, to 100*–highly attracted to*. We reverse-coded responses to the second item (attraction to men) and summed both responses, with total scores ranging from 0–200. Higher scores on the scale reflect exclusive attraction towards different-gender partners (i.e., women), and lower scores reflect exclusive attraction toward same-gender partners (i.e., men). An inspection of the distribution of sexual attraction scores showed a clear bimodal distribution (Figure 2). A further examination revealed that 80% of participants fell below 10 or above 190 on our scale, indicating our sample was mainly exclusively attracted to either women or men. We, therefore, categorised our participants into same-gender (*n* = 86) and different-gender attraction (*n* = 87) by splitting along the scale midpoint (i.e., 100)^3^3We acknowledge the limitations of dichotomising a continuous measure. However, the clear bimodal distribution made this necessary. For prudence, we ran our models, including sexual attraction as a continuous measure and found little difference from those using the categorised data below. We present these findings in Cahill et al. (2023, 2024)..

#### Body Dissatisfaction

We measured body dissatisfaction using the 24-item Male Body Attitudes Scale (MBAS; Tylka et al., 2005). The scale comprised three 8-item subscales: weight (e.g., “*I think my body should be leaner*”), muscularity (e.g., *“I have too little muscle on my body”*), and height (e.g., *“I wish I were taller”*). Participants responded on a 1–*Never* to 7–*Always* scale. We summed participant’s responses to the 24 items to form a scale score where higher scores reflected greater body dissatisfaction.

#### Quality of Life

We measured quality of life with the 26-item World Health Organisation Quality of Life Scale–Abbreviated (WHOQOL-BREF; Skevington et al., 2004). We used 24 items^4^4We excluded two single item measures asking about overall perceptions of quality of life as were interested in the association between sexual attraction, body dissatisfaction and each domain of quality in life. to assess quality of life across four domains: physiological (7–items; e.g., *“To what extent do you feel that physical pain prevents you from doing what you need to do?”*), psychological (6–items; e.g., *“How much do you enjoy life?”*), social (3–items, e.g., *“How satisfied are you with your personal relationships?”*), and environmental (8–items; e.g., *“How safe do you feel in your daily life?”*). Participants responded on 1–*Not at all/Very Poor* to 7–*Completely/Very Good* scales. We summed the relevant items to create scale scores for the four domains of quality of life. Higher scores reflected greater self-reported quality of life (i.e., more positive).

### Data Analysis

The data and R scripts are at Cahill et al. (2023). Before completing our primary analyses, we investigated whether there was a correlation between age and body dissatisfaction and age and quality of life in our sample. This check was necessary as we identified a significant age difference between men reporting the same gender and different gender attraction, *t*(171) = 2.65, *p* = .009, Cohen’s *d* = 0.41, 95% CI [0.10, 0.71]. However, age did not correlate with either body dissatisfaction or any domain of quality of life. We, therefore, decided not to control for age within our subsequent analyses.

We used OLS regression to test our first three hypotheses, using standardised predictor and outcome variables. We tested Hypothesis 4 by testing the indirect effect of sexual attraction via body dissatisfaction on the four quality of life domains. Sexual attraction was a dummy coded variable (0, 1; same, different). We tested these indirect effects using a product-of-coefficients approach (Yzerbyt et al., 2018). We quantified the uncertainty around the indirect effects using 95% bias-corrected bootstrapped confidence intervals using 10,000 bootstrap samples.

## Results

First, we tested whether self-reported sexual attraction influences quality of life. Sexual attraction predicted *psychological* quality of life, β = .48, 95% CI [.18, .77], *t*(171) = 3.21, *p =* .002, Cohen’s *d* = 0.49, 95% CI [0.19, 0.79]. As expected, men who reported attraction to men reported lower psychological quality of life (*M* = 25.13, *SD* = 7.48) than men who reported attraction to women (*M* = 28.60, *SD* = 6.73). Sexual attraction was not associated with *physiological* quality of life, β = .13, 95% CI [-0.17, 0.43], *t*(171) = 0.86, *p* = .393, Cohen’s *d* = 0.13, 95% CI [-0.17, 0.43], same gender attraction: *M* = 37.95, *SD* = 8.39; different gender attraction: *M* = 38.97, *SD* = 7.11), *social* quality of life, β = .22, 95% CI [-0.08, 0.52], *t*(171) = 1.44, *p =* .153, Cohen’s *d* = 0.22, 95% CI [-0.08, 0.52], same gender attraction: *M* = 12.71, *SD* = 4.66; different gender attraction: *M* = 13.70, *SD* = 4.42, or *environmental* quality of life, β = .11, 95% CI [-0.19, 0.41], *t*(171) = 0.70, *p* = .485, Cohen’s *d* = 0.11, 95% CI [-0.19, 0.41], same gender attraction: *M* = 40.13, *SD* = 9.08; different gender attraction: *M* = 41.07, *SD* = 8.61). In summary, we found that sexual attraction (i.e., being attracted to men) was only associated with psychological quality of life but not physiological, social, or environmental quality of life.

Second, we regressed MBAS scores (i.e., body dissatisfaction) on sexual attraction. Sexual attraction was a significant predictor for MBAS scores, β = -.37, 95% CI [-0.67, -0.08], *t*(171) = -2.49, *p =* .014, Cohen’s *d* = 0.38, 95% CI [0.08, 0.68]. Indeed, men who reported attraction to men displayed greater body dissatisfaction (*M* = 107.01, *SD* = 29.82) than men who reported attraction to women (*M* = 96.35, *SD* = 26.52).

Third, we regressed each quality of life domain on MBAS scores while controlling for sexual attraction (Table 2). Results showed negative associations between body dissatisfaction and each quality of life domain while controlling for sexual attraction.

**Table 2:** MBAS Scores Predicting Each Quality of Life Domain While Controlling for Sexual Attraction

Predictor	β	*SE*	*t*	*p*
*Psychological QoL*				
MBAS	-.38 [-.50, -.25]	.07	-5.38	< .001
Sexual Attraction_(same/different)_	.34 [.08, .61]	.14	2.40	.018
*Physiological QoL*				
MBAS	-.32 [-.45, -.18]	.07	-4.25	< .001
Sexual Attraction_(same/different)_	.01 [-.27, .30]	.01	0.09	.926
*Social QoL*				
MBAS	-.31 [-.44, -.17]	.07	-4.23	< .001
Sexual Attraction_(same/different)_	.10 [-.18, .40]	.15	0.69	.491
*Environmental QoL*				
MBAS	-.32 [-.45, -.19]	.07	-4.31	< .001
Sexual Attraction_(same/different)_	-.01 [-.30, .27]	.15	-0.08	.933

*Note*. Outcome in italics. QoL = quality of life. Numbers in brackets represent 95% confidence intervals. The *df* for each test was 170.

Finally, we explored each indirect effect. We found significant indirect effects (i.e., the confidence interval excluded 0) of sexual attraction via body dissatisfaction on *psychological* quality of life, β = .14, *SE = .*06, 95% CI [0.04, 0.29], *physiological* quality of life, β = .12, *SE* = .06, 95% CI [0.03, 0.25], *social* quality of life, β = .12, *SE* = .06, 95% CI [0.03, 0.25], and *environmental* quality of life, β = .12, *SE* = .06, 95% CI [0.03, 0.26].

## Discussion

Using a cross-sectional design, cisgender men reported their body dissatisfaction, quality of life (psychological, physiological, social, and environmental), and sexual attraction toward men and women. Contrasting previous work, we measured men’s self-reported sexual attraction, a dimension of sexual identity that is frequently understudied.

Our research investigated the association between sexual attraction, body dissatisfaction and quality of life in men across their psychological, physiological, social, and environmental quality of life domains. We also explored whether men attracted to men would report lower quality of life and whether body dissatisfaction mediates the association between sexual attraction and quality of life. Our findings indicate that men attracted to men report higher body dissatisfaction and lower psychological quality of life than men attracted to women. Additionally, body dissatisfaction in men was associated with lower quality of life across all domains. These findings support our first two hypotheses. We found partial support for Hypothesis 3: men attracted to men showed significantly lower psychological quality of life but not lower quality of life in the other domains. Finally, supporting Hypothesis 3, we found significant indirect effects of sexual attraction through body dissatisfaction on all quality of life domains.

Previous research has shown that sexual identity, when measured using categorical identifiers (e.g., selecting gay or straight), is associated with body dissatisfaction. Men who report being gay displayed greater body dissatisfaction than straight men (Dahlenburg et al., 2020). However, sexuality is multidimensional, and other dimensions (e.g., sexual attraction) have not been explored within the literature (He et al., 2020). Our findings are novel and show that these associations generalise to other dimensions of sexual identity, specifically sexual attraction toward men and women. Future research could expand our findings by exploring each dimension simultaneously to test for unique associations between sexual identity dimensions and body dissatisfaction.

Our findings support the position that body dissatisfaction may increase when a person is attracted to and aims to attract men (Ehlinger & Blashill, 2016). This trend is because physical attractiveness among men is central to acquiring sexual partners (Frederick & Essayli, 2016). This may explain why straight women also report higher body dissatisfaction, whereas lesbians do not (Alvy, 2013). By internalising the strict appearance standards that men perpetuate, men attracted to men may experience self-objectification, leading to increased body surveillance and dissatisfaction (Brewster et al., 2017; Wood, 2004). Furthermore, as men attracted to men occupy a minoritised status, they likely experience similar pressures surrounding their body image to self-identifying gay men (Frederick & Essayli, 2016; Rodgers et al., 2023). Specifically, they likely experience appearance discrimination and societal pressures to adopt a traditionally masculine body type (Brewster et al., 2017). These factors may explain the commonality in findings between previous research categorising identity using self-identifiers and our findings using self-reported sexual attraction. Interventions directed toward improving body dissatisfaction should account for individuals most at risk. Here, we show that men attracted to men are one such population.

We also found that body dissatisfaction was associated with lower psychological, physiological, social, and environmental quality of life in men. This supports the notion that body dissatisfaction is associated with quality of life in men (Griffiths et al., 2016, 2019). However, we also expand this by showing that, like women, men also experience consequences of body dissatisfaction and reduced quality of life across each domain (Santhira Shagar et al., 2021). Our findings support that body dissatisfaction is associated with a range of negative behaviours, which can impair quality of life across each domain (Mond et al., 2013). For example, body dissatisfaction can negatively impact self-worth and mood, cause unhealthy and obsessive physical activities to improve their physique (e.g., excessive dieting or exercise), and negatively impact social relationships by causing appearance competitions (Lawler & Nixon, 2011; Tylka, 2011). Given that having an ideal body is perpetuated within various forms of media and men are often pressured to conform to this ideal, this may cause men to view their immediate environment as hostile and confrontational (Tylka, 2011).

Our findings emphasise the potential subclinical negative influence of body dissatisfaction in men. It extends the current evidence base, primarily focusing on body dissatisfaction’s adverse clinical and pathological outcomes. We add to the growing body of literature emphasising that body dissatisfaction is a global public health concern by illustrating the potential subclinical outcomes for men (Griffiths et al., 2017; Rodgers et al., 2023). Our findings also support the practical notion that interventions should counter body dissatisfaction before a person’s symptoms reach clinical significance and not after due to the ongoing adverse effects on health and well-being (Diedrichs et al., 2021).

As noted above, we found only partial support for the prediction that men attracted to men would report reduced quality of life across each domain relative to men attracted to women. Specifically, we found that men attracted to men experienced poorer psychological quality of life than men who reported sexual attraction to women. Minority Stress Theory may help explain these findings; minoritised groups (e.g., sexual minoritised men) experience increased pressures to conform to societal norms, resulting in impaired psychological well-being (Grabski et al., 2019; Meyer, 2003). However, we did not find differences in physiological, social, or environmental quality of life between either reported sexual attraction pattern. This finding suggests that the additional pressures men attracted to men face (e.g., ostracisation; Morgenroth et al., 2022) may not negatively influence all aspects of their daily well-being and may specifically influence psychological processes.

Although our results indicated no direct association between sexual attraction and physiological, social, or environmental quality of life, there were significant indirect effects between sexual identity as defined by sexual attraction quality of life across each domain, which acted through body dissatisfaction. As only psychological quality of life was directly associated with sexual attraction, we are reluctant to interpret the indirect effects for the remaining domains. Men attracted to men experienced lower psychological quality through body dissatisfaction, which is plausible given that body dissatisfaction is associated with poor self-esteem and low mood (Barnes et al., 2020; Blashill, 2011). Men who are attracted to men also experience unique negative experiences concerning their body image, such as greater pressure from society and potential partners to be attractive and to have a body ideal that is difficult to attain and more frequent instances of objectification from others (Brewster et al., 2017; Tiggemann et al., 2007). In turn, these experiences and the negative emotional and cognitive processes associated with body dissatisfaction in men attracted to men facilitate reductions in psychological quality of life.

This finding emphasises that specific groups in society may be more at risk of poorer health and well-being because of their body dissatisfaction, and policies designed to tackle body dissatisfaction should be conscious of these populations. Potentially, one psychological process that may negatively influence psychological well-being can be mitigated by reducing body dissatisfaction in these at-risk groups. Interventions to improve quality of life men attracted to men could focus on improving body satisfaction. Effective interventions are especially important considering the wide range of stressors that men not adhering to traditional heteronormative ideals experience (e.g., discrimination), and the high rates of psychological distress and suicidality in sexual minoritised men generally (Chen et al., 2020; ILGA, 2020; Marshal et al., 2011).

### Limitations and Suggestions for Future Research

Our research has several limitations. First, we focused on exploring men’s sexual identity as defined by self-reported attraction patterns. While this was a strength and deviated from using categories (i.e., straight or gay), we did not account for the intersectional nature of sexual identity. Namely, sexual identity consists of identity, attraction and behavioural components, and future research may wish to account for the intersection between these components (e.g., men who identify as straight but report attraction to other men; He et al., 2020; Kaestle, 2019). Second, we restricted our sample to primarily white participants from the UK and Ireland. While this was necessary to avoid potential cultural confounds, given that body ideals vary based on ethnicity and cultural identity (Paxton et al., 2006), this limits our findings’ generalisability to white, Western populations only. Third, our coefficient for the association between sexual attraction and body dissatisfaction was slightly below the minimum effect size that our study could reliably detect (Cohen’s *d* = 0.38 vs. a minimum detectable effect size of .43). Future research should replicate our finding using the effect we report in an a priori power analysis. All other effects we interpreted exceeded our study’s minimum detectable effect size. Finally, though compelling, our data are correlational, so we cannot make conclusive claims about causality between the identified associations.

We showed that men attracted to men experience lower quality of life, potentially due to body dissatisfaction. Thus, identifying effective interventions is paramount. As we outlined, men attracted to men, like self-identifying gay men, are more likely to endorse societal body ideals and experience pressure to be attractive from partners (Brewster et al., 2017; Frederick & Essayli, 2016). Future research could determine if the internalisation of societal ideals and appearance pressures from partners mediates the association between sexual attraction and body dissatisfaction, as argued here. If so, potential interventions could target these processes by increasing resilience to external appearance pressures.

### Conclusion

Our study demonstrated that men who report attraction to men show greater body dissatisfaction and reduced psychological quality of life than those who report attraction to women. We also found that men’s body dissatisfaction was not only associated with reduced quality of life across each domain but also mediated the relationship between sexual attraction and quality of life. Our findings emphasise the global public health problem associated with body dissatisfaction and that specific groups may be more at risk of poorer health and well-being due to body dissatisfaction.

## Data Availability

The data and R script that support the findings of this study are openly available at Cahill et al. (2023).
